# High-quality draft genomes of ecologically and geographically diverse *Psilocybe* species

**DOI:** 10.1128/mra.00250-24

**Published:** 2024-12-27

**Authors:** Ian M. Bollinger, Harte Singer, Jordan Jacobs, Marshall Tyler, Kelsey Scott, Christopher S. Pauli, Dusty Rose Miller, Caine Barlow, Alan Rockefeller, Jason C. Slot, Victor Angel-Mosti

**Affiliations:** 1 Entheome Foundation, Oakland, California, USA; 2 TrypLabs LLC, Portland, Oregon, USA; 3 Field Trip Natural Products, Kingston, Jamaica; 4 Ohio State University, Columbus, Ohio, USA; 5 Tryptomics LLC, Longmont, Colorado, USA; 6 Vanderbilt University, Nashville, Tennessee, USA; 7 Entheogenesis Australis, Belgrave, VIC, Australia; 8 Counter Culture Labs, Oakland, California, USA; 9 Department of Plant Pathology, The Ohio State University, Columbus, Ohio, United States; 10 Center for Psychedelic Drug Research and Education, The Ohio State University, Columbus, Ohio, United States; University of Maryland School of Medicine, Baltimore, Maryland, USA

**Keywords:** *Psilocybe*, genome, psilocybin, psilocybin gene cluster, hybrid assembly, Illumina, Oxford Nanopore Technologies

## Abstract

*Psilocybe* is a genus of mushroom-forming fungi with ecological, ethnomycological, and clinical importance due to psilocybin production by most species. We present five genomes that enable deeper discovery and analysis of the psilocybin gene cluster and increase taxonomic resolution within *Psilocybe*: *Psilocybe semilanceata*, *Psilocybe gandalfiana* nom. prov., *Psilocybe caeruleorhiza*, *Psilocybe azurescens*, and *Psilocybe allenii*.

## ANNOUNCEMENT

*Psilocybe* is a genus of mushroom-forming Basidiomycota fungi with ecological, ethnomycological, and clinical importance due to psilocybin production by most of its species (1). Of the ~165 recognized *Psilocybe* species, only eight were previously represented by whole genomes on GenBank (2). Past genomic studies of *Psilocybe* (3) investigated cultivated and native populations of *Psilocybe cubensis* (4, 5), *Psilocybe subaeruginosa* (6), and herbarium metagenomes (2). Here, we present five new genomes from wild-collected, ecologically diverse *Psilocybe* (Table 1) to inform the taxonomy, ecology, and metabolic diversity of the genus.

**TABLE 1:** Species information and assembly statistics

	*Psilocybe semilanceata*	*Psilocybe gandalfiana* nom. prov.	*Psilocybe caeruleorhiza*	*Psilocybe zapotecorum* (7)	*Psilocybe azurescens*	*Psilocybe allenii*
Organism data						
iNaturalist/ Mushroom Observer#	iN: 141588727	MO: 494175	iN: 160500191	iN: 36797577	iN: 36611799	iN: 100444475; iN: unavailable
Geographic location	Clatsop County Coast, OR, USA	Stanislaus National Forest, CA, USA	Pittsburgh Region, PA, USA	La Martinica, Veracruz, Mexico	East San Francisco Bay, CA, USA	Napa-Sonoma- Russian River Valleys, CA, USA; unavailable
Substrate	Soil; open grassy meadow	Soil; open grassy meadow	Soil; hillside beneath leaf litter and woodchips	Soil; grassy road near river beneath oak	Soil; beneath redwood leaf litter	Soil; beneath oak leaf litter
Karyology	Monokaryon	Monokaryon	Dikaryon	Dikaryon	Dikaryon	Dikaryon, dikaryon
BioSample accession number	SAMN37293870	SAMN39911971	SAMN39911972	SAMN37305711	SAMN39911973	SAMN39911974
Raw data statistics						
Illumina SRRs	SRR25932370_1; SRR25932370_2	SRR27945397_1; SRR27945397_2	SRR27945395_1; SRR27945395_2	SRR25932370_1; SRR25932370_2	SRR27945393_1; SRR27945393_2	SRR27945391_1; SRR27945391_2
Illumina total reads	24,794,826	24,063,937	25,242,672	24,794,826	25,153,451	27,636,688
Illumina depth	96.3×	78.9×	63.4×	63.7×	47.3×	54.8×
ONT SRR	SRR25920759	SRR27945396	SRR27945394	SRR25932369	SRR27945392	SRR27945390
ONT total reads	764,901	1,037,152	537,056	1,332,184	1,303,074	3,125,548
ONT *N*_50_	9,368.0	8,313	9,058	9,164	8,749	3,899
Hybrid assembly statistics						
Genomic assembly	GCA_040207485.1	GCA_040207425.1	GCA_040207415.1	GCA_040207405.1	GCA_040207365.1	GCA_040207355.1
Genome size (bp)	38,605,746	45,751,632	59,770,098	58,364,877	79,795,199	75,618,805
Number of contigs	41	158	2,521	1,162	2,305	2,885
*N*_50_ (bp)	2,734,957	1,649,840	64,270	173,255	110,549	113,222
Contig *L*_50_	6	11	270	71	200	172
GC content (%)	47.08	46.44	46.23	46.58	45.70	45.70
Agaricales BUSCOs (*n* = 3,870), Basidiomycota BUSCOs (*n* = 1,764)						
Complete	**99.0%, 99.8%**	**99.3%, 99.4%**	**99.1%, 99.6%**	**99.0%, 99.7%**	**99.2%, 99.6%**	**99.0%, 99.5%**
Single	99.0%, 99.7%	98.9%, 99.0%	93.0%, 93.8%	93.9%, 93.7%	92.3%, 92.5%	92.5%, 92.6%
Duplicated	0.1%, 0.1%	0.3%, 0.4%	6.2%, 5.7%	5.1%, 6.0%	7.0%, 7.1%	6.6%, 6.8%
Fragmented	0.0%, 0.1%	0.0%, 0.1%	0.1%, 0.3%	0.1%, 0.2%	0.3%, 0.3%	0.3%, 0.3%
Missing	0.9%, 0.2%	0.7%, 0.5%	0.8%, 0.2%	0.9%, 0.2%	0.5%, 0.1%	0.7%, 0.2%

^*a*^ Bold values represent quick references for species names and their associated BUSCO values as a means of highlighting the "High-Quality" properties for each species.

The type species, *Psilocybe semilanceata* (8), is widely distributed in European, Australian, and Northern and Southern American temperate grasslands. *Psilocybe allenii* (9) and *Psilocybe azurescens* (10) are members of the wood-decomposing Section Subaeruginosae (3) found in Australia, Aotearoa, and coastal Northwest America (11). *Psilocybe caeruleorhiza* (12) is a riparian wood decayer from the Eastern US (12). *Psilocybe gandalfiana* nom. prov., newly found in the Western US, currently undescribed, produces a secotioid sporocarp, decays wood, and fruits from soil.

A *P. caeruleorhiza* dikaryon and *P. semilanceata* and *P. gandalfiana* monokaryons were grown from spore. *P. allenii* and *P. azurescens* were cloned from fruit body: whole sporocarps were wild collected; 2 mm sterile squares were incubated on 2% malt extract agar until mycelium grew. Culture identities were confirmed using morphology and nuclear ITS sequence. Isolates were broth-cultured (21 days), filtered, washed, and homogenized before genomic DNA Extraction (DNeasy Plant Mini Kit; CAT No. 69104). Short-read DNA libraries (NEBNext Ultra DNA library preparation kit; CAT No. E7370L) were sequenced with 150-bp paired-end format on the NovaSeq (Illumina) by Novogene returning Q30 reads or better for 85% of the data. All isolates were sequenced by Novogene at 100× depth Nanopore sequencing using a PromethION system (Oxford Nanopore Technologies [ONT]), except *P. allenii*, which was sequenced using MinION (ONT) with the SQK-LSK112 ligation sequencing kit (CAT No. 69104) to >73× depth. All isolates used R10 flow cells in ONT sequencing without shearing or size selection. Default parameters for software were used except when noted in brackets. Illumina reads were trimmed with Trimmomatic version 0.36 (13) (PE-phred33,ILLUMINACLIP TruSeq3-PE.fa:2:30:10:11,HEADCROP 10, CROP 145,SLIDINGWINDOW 50:25,MINLEN 125). MinION and PromethION reads were Guppy version 3.0.3 (14) and Dorado 7.2.13 (15) base called, respectively. Reads were quality checked with FastQC (16) and NanoPlot version 1.43.0 (17) (Table 1). Raw ONT reads were *de novo* assembled with Flye version 2.9.2 (18) (–nano-hq,–keep-haplotypes). Long reads were mapped to the assembly with BWA version 0.7.17 (19). An indexed, sorted BAM file was generated with samtools version 1.19 (20). Hybrid assemblies were constructed in Pilon version 1.24 (21) using the BAM file, trimmed Illumina reads, and ONT Flye *de Novo* Assemblies (--changes,--vcf,--diploid). BUSCO orthologs (22) were benchmarked with compleasm version 0.2.5 (23), referencing Agaricales and Basidiomycota (OrthoDB v10) data sets (22). Psilocybin biosynthesis gene coordinates were extracted using exonerate version 2.2.0 (24) with reference to *P. cubensis* (25) cluster sequences (--model protein2genome:bestfit,--maxintron 100,--percent 0.2,--showalignment yes,--showtargetgff yes,--showvulgar no,-E). Gene cluster loci were aligned with clinker version 0.0.30 and clustermap version 0.0.0 (11, 26) (-i 0.3).

**Fig 1:**
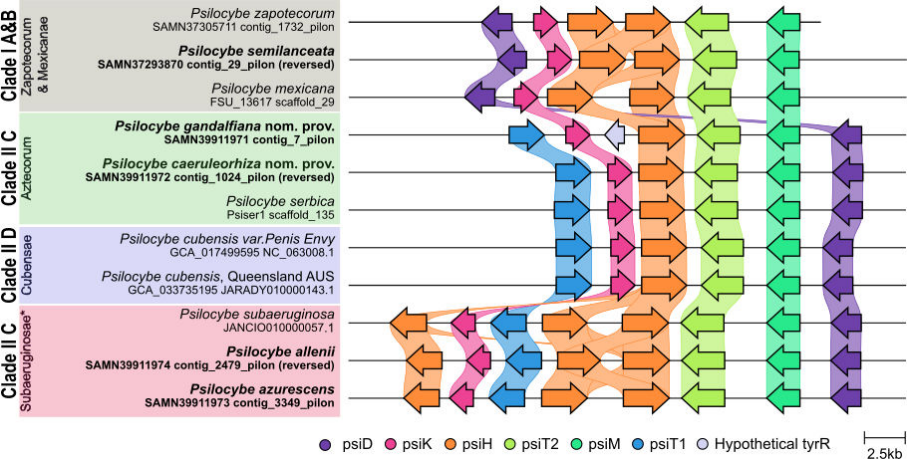
Psilocybin gene clusters across species. Current genomes are in bold showing high alignment with previously reported genomes. The alignment shown suggests that Clade II C may be two separate groups. Section designations are shown under the clade designations (i.e., Cubensae). The hypothetical tyrR gene observed in *P. gandalfiana* (white).

The draft genomes of ecologically and geographically diverse *Psilocybe* species were deemed high quality based on the recovery of full psilocybin clusters, high BUSCO recovery, and *N*_50_. Organism data, information on the ecology and locality as well as raw data statistics and hybrid assembly statistics are detailed in Table 1. Genomes ranged from ~39 to 80 Mbp. *Psilocybe zapotecorum*, announced previously, is included in Table 1 for reference (7). Assembly completeness was >98.8% BUSCO recovery with Agaricales and Basidiomycota; no incomplete BUSCO genes were observed. Psilocybin clusters largely matched the described synteny: *P. gandalfiana, P. caeruleorhiza, P. allenii,* and *P. azurescens* matched *P. subaeruginosa* (27), whereas *P. semilanceata* was similar to *Psilocybe mexicana* (27) (Fig. 1). A novel hypothetical tyrosine recombinase (tyrR) was observed between psiK and psiH in *P. gandalfiana*.

## Data Availability

Raw data and assemblies are in GenBank BioProject PRJNA1013220, and accession numbers are in Table 1.
